# Supplementary material for: Synergistic Pro-Apoptotic Effect of a Cyclic RGD Peptide-Conjugated Magnetic Mesoporous Therapeutic Nanosystem on Hepatocellular Carcinoma HepG2 Cells
Source: Pharmaceutics. 2023 Jan 13;15(1):276. doi: 10.3390/pharmaceutics15010276 (PMC9866545; doi:10.3390/pharmaceutics15010276)
Supplement: Supplementary file 1 [file pharmaceutics-15-00276-s001.zip › pharmaceutics-2129308-supplementary.pdf]

Supplementary Information

Synergistic pro-apoptotic effect of cyclic RGD peptide-conjugated magnetic mesoporous therapeutic nanosystem on hepatocellular carcinoma HepG2 cells

Xuanping Zhao [1,2], Chuan Liu [1,2], Zichao Wang [1,2], Yingyuan Zhao [1,2], Xuyang Chen [1,2], Haizhen Tao [1,2], Hong Chen [1,2], Xueqin Wang [1,2,]* and Shaofeng Duan [3,]*

1 College of Bioengineering, Henan University of Technology, Zhengzhou 450001, China; 18239703962@163.com (X.Z.); lc18772858256@163.com (C.L.); 6832917@163.com (Z.W.); anxiaoying2010@163.com (Y.Z.); chenxuyang1013@163.com (X.C.); tm771066547@163.com (H.T.); ch1061265684@163.com (H.C.)

2 Key Laboratory of Functional Molecules for Biomedical Research, Henan University of Technology, Zhengzhou 450001, China

3 Institute for Innovative Drug Design and Evaluation, School of Pharmacy, Henan University, Kaifeng 475004, China

* *Correspondence: wangxq0708@163.com (X.W.); sduan@henu.edu.cn (S.D.); Tel.: +86-371-67756928 (X.W.); +86-371-22822134 (S.D.)*

This supporting information provides all of the additionalinformation as noted in the manuscript and more detailed discussion of the current study.

Synthesis of SPIO NPs.SPIO NPs i.e., γ-Fe_2O_3 NPs used in this study were synthetized from magnetite (Fe_3O_4) according to the method reported previously proposed elsewhere[1]. First, 4.5 mL $FeCl_3$ (2 M in 2 M HCl) was added to 15.5 mL DI water, followed by the addition of 3 mL Na_2SO_3 (1 M) within 1 min under stirring. When the color of the solution changed from red to light yellow, it was added to 120 mL of NH_4OH solution (0.85 M) with vigorous stirring. A black precipitate quickly formed and was allowed to crystallize completely for another 40 min. After washing with deoxygenated water, the black precipitate was diluted to 252 mL (with a mass concentration of 3 mg/mL) and then adjusted to pH 3.0 with HCl (0.1 M). The suspension was then heated to 90 °C in 5 min, and was stirred under aeration (with air) for 90 min at 110°C. The color of the suspension slowly changed from black to reddish-brown. After washing with DI water by magnetic decantation, the reddish-brown precipitate was dried to afford SPIO NPs as a powder.

Preparation of SPIO@MSN NPs. The SPIO@MSN NPswere synthesized utilizingsurfactanttemplatedseeded-growth method[2].Briefly, 1.48mL SPIO NPs in chloroform (6.1 mg/mL) was added to 10 mL aqueous CTAB (0.08 M), followed by ultrasonication for 30min to removethe chloroform. The dispersion solution was added to a mixturecomposed of 60 mL water, 20 mL ethylene glycol, and 1.4mL NH_4OH, and thenincubatedfor 10 min at 70°C.Subsequently, 1.4mL decane was added to the mixture and stirred for 30 min, followed by the addition of 340 μLTMB for 120 minofhomogenization.After that, 1 mL TEOS was added and the reaction was continued at 70°Cfor 180 min. Then, theprepared colloidal solution was centrifuged at 10,000 rpmfor 20 min, and thesurfactant CTAB wasremovedby ion exchange. Next, the prepared MSN wasdispersedin 80 mL ethanol containing 80 mg NH_4NO_3 and stirred for at 60°C120 min.Finally, the resulting nanoparticles were washed using ethanol and dried at 60°Cundervacuumovernight.

Characterization. Transmission electron microscopy (TEM, Ruli HT7700) was used toanalyze the morphology of the nanoparticles, and Fourier transforms infrared spectroscopy (FT-IR, Nicolet IS10) was used to investigate the structuralcomposition of the fabricated nanocomposites. The particle size and potential distribution of nanocomposites were measured by Malvern laser particle sizer (Nano ZS90).The crystal structuresof nanocomposites were examinedby X-ray Diffraction (XRD, X'Pert PRO MPD, Holland Panalytical). The specific surface area and pore size of the nanocomposites were analyzed by the nitrogen adsorption-desorption experiment.The absorbance was measured using a UV-visible spectrophotometer (UV-3100PC) and microplate spectrophotometer (Tecan Spark).

Statistical analysis. Bright-field and fluorescence images were captured using an inverted fluorescence microscope (Axio Observer 3).Software Image-Pro Pluss 6.0 (IPP)was used to perform image analysis. All the dataarepresented as mean ±standard deviation (SD). *p< 0.05 was considered significant, **p< 0.01 was considered moderately significant, and ***p< 0.001 was considered highly significant. Data were collected by flow cytometry (BD FACS Calibur) and analyzed with Cell Quest Pro software.

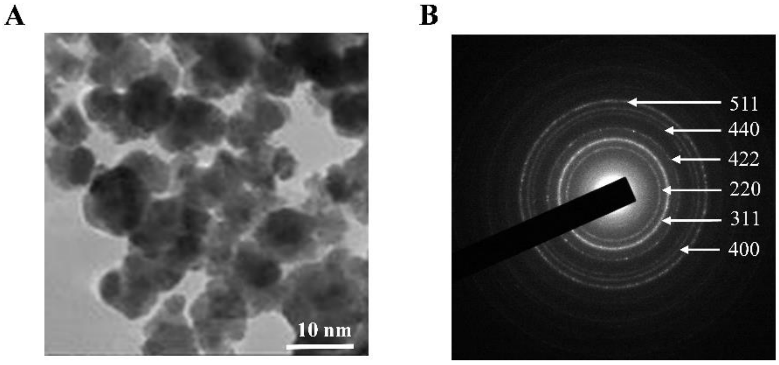

Figure S1. TEM image(A) and ED pattern(B) of γ-Fe_2O_3 NPs.

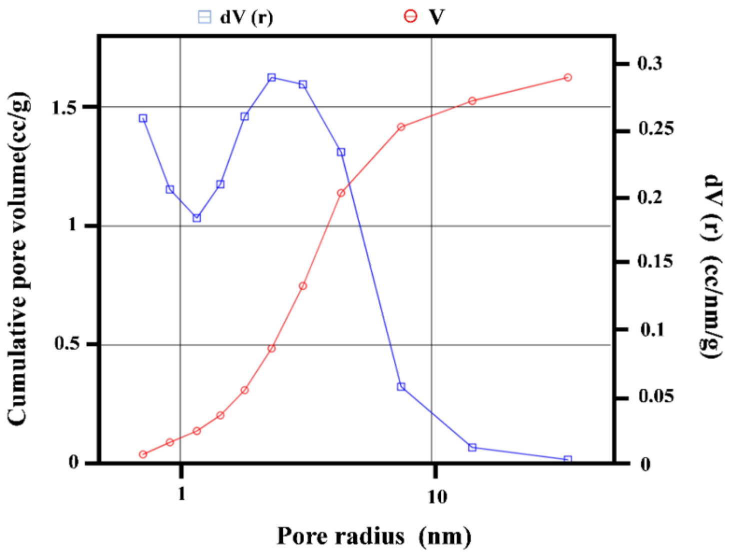

Figure S2. Pore Volume vs Cumulative Volume Statistics Plot of SPIO@MSN NPs.

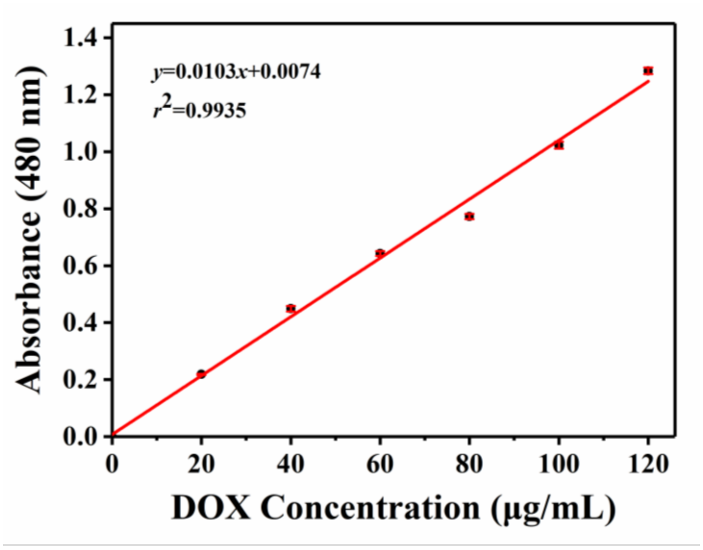

Figure S3. Standard curve of DOX.

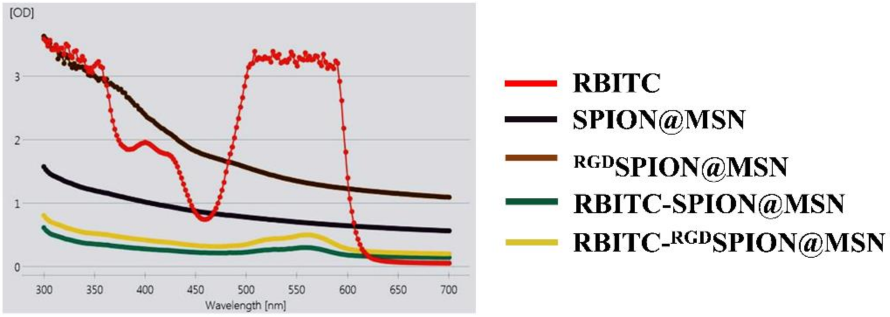

Figure S4. UV absorption spectrum of RBITC-labeled SPIO@MSN NPs and^{RGD}SPIO@MSN NPs.

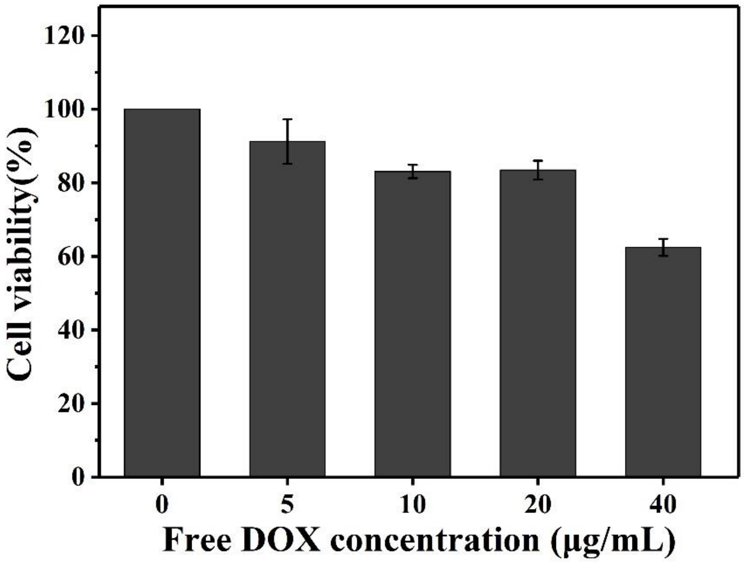

Figure S5. Assay of cell viability treatment with free DOX for 24 h.

References

1. Qu, S.; Yang, H.; Ren, D.; Kan, S.; Zou, G.; Li, D.; Li, M. Magnetite nanoparticles prepared by precipitation from partially reduced ferric chloride aqueous solutions. *J. Colloid Interface Sci.* ***1999****, 215*, 190–192.
2. Wang, X.; Xiong, T.; Cui, M.; Guan, X.; Yuan, J.; Wang, Z.; Li, R.; Zhang, H.; Duan, S.; Wei, F. Targeted self-activating Au-Fe_3O_4 composite nanocatalyst for enhanced precise hepatocellular carcinoma therapy via dual nanozyme-catalyzed cascade reactions. *Appl. Mater. Today.* ***2020****, 21*, 100827
